# An Unusual Presentation of a Small Bowel Injury in an Adult Following a Fall From Height

**DOI:** 10.7759/cureus.80972

**Published:** 2025-03-21

**Authors:** Shrikant Manwatkar, Preksha Rani, Narendra Chaudhary, Biplab Mishra

**Affiliations:** 1 Trauma and Critical Care, Command Hospital Air Force, Bengaluru, IND; 2 Trauma Surgery and Critical Care, All India Institute of Medical Sciences, New Delhi, IND; 3 Surgery, All India Institute of Medical Sciences, New Delhi, IND; 4 Trauma and Acute Care Surgery, All India Institute of Medical Sciences, New Delhi, IND

**Keywords:** blunt trauma abdomen, inflammatory mass, intestinal obstruction, intestinal perforation, trauma

## Abstract

Blunt trauma to the abdomen (BTA) poses a challenge to surgeons in diagnosing the exact intra-abdominal injuries, including hollow viscus injuries. This case report discusses a 40-year-old male patient, a case of BTA following a fall from a height, who did not present any features of peritonitis. Upon evaluation, the patient was found to have a small bowel obstruction in the distal jejunum. The patient was taken for exploratory laparotomy, and the inflammatory mass was removed. A side-to-side distal jejunal anastomosis was performed. This case report teaches us that not all small bowel injuries present as perforation peritonitis; they can also manifest as intestinal obstruction. Delays in diagnosis and treatment can lead to increased morbidity and complications.

## Introduction

Trauma significantly contributes to morbidity and mortality among young people in developing countries [1]. Abdominal injuries primarily result from road traffic accidents [2]. The abdomen is the third most commonly injured body region [3]. Most abdominal injuries are blunt in nature and are managed nonoperatively, with approximately 25% of these injuries requiring surgical intervention. The spectrum of injuries involving the small bowel in blunt trauma ranges from contusions and mesenteric hematomas to obvious perforations or lacerations, collectively accounting for about 4%-15% [4]. Bowel contusions and mesenteric hematomas make up the majority of these injuries; however, small bowel perforations occur in less than 1% of patients with blunt abdominal trauma (BTA) [5]. Additionally, acute small bowel obstruction is a rare feature of BTA.

Significant emphasis is placed on conducting a meticulous clinical examination to rule out even the most subtle abdominal injuries. However, clinical examination alone may not be sufficient among polytrauma patients with altered mental status harboring head injury. There are high chances of missed intra-abdominal injury with associated morbidity and mortality, especially in patients who survive the initial phase following an injury. Contrast-enhanced computed tomography (CECT) of the abdomen is the recommended diagnostic method for patients exhibiting clinically subtle or undetectable signs of abdominal injury. Many polytrauma patients are referred from peripheral healthcare facilities after significant delays in evaluation and definitive intervention. This delayed diagnosis progressively worsens the patient’s condition over time, undermining their ability to heal and recover. Morbidity and mortality rates remain high in these patients despite the latest techniques and advancements in diagnostic and supportive care.

We encountered a patient who sustained BTA and presented with clinical features suggestive of intestinal obstruction rather than common features of mesenteric hematoma or perforation peritonitis following small bowel injury. There was a delay in definitive diagnosis and management, as the patient was only able to reach the Level I Apex Trauma Center 17 days after injury after a 2,280 km train journey. Due to the delayed diagnosis, even after undergoing exploratory laparotomy, the patient developed a burst abdomen and respiratory distress during the postoperative period.

## Case presentation

A male patient in his 40s sustained a BTA following a fall from approximately 50 feet in the distant mountains. The patient was admitted to a nearby medical college with complaints of abdominal pain. A CECT of the abdomen revealed a grade II splenic injury with minimal hemoperitoneum. The patient was managed non-operatively for this condition. The patient remained symptomatic with abdominal pain and later developed obstipation. The patient continued to be managed nonoperatively but did not improve. After 15 days following the initial injury, the patient was referred to a level I Apex Trauma Centre for further management. The patient traveled 2,280 km by train, taking three days to reach the level I trauma center. The patient was admitted to the level I trauma center under the Division of Trauma Surgery and Critical Care.

**Figure 1:**
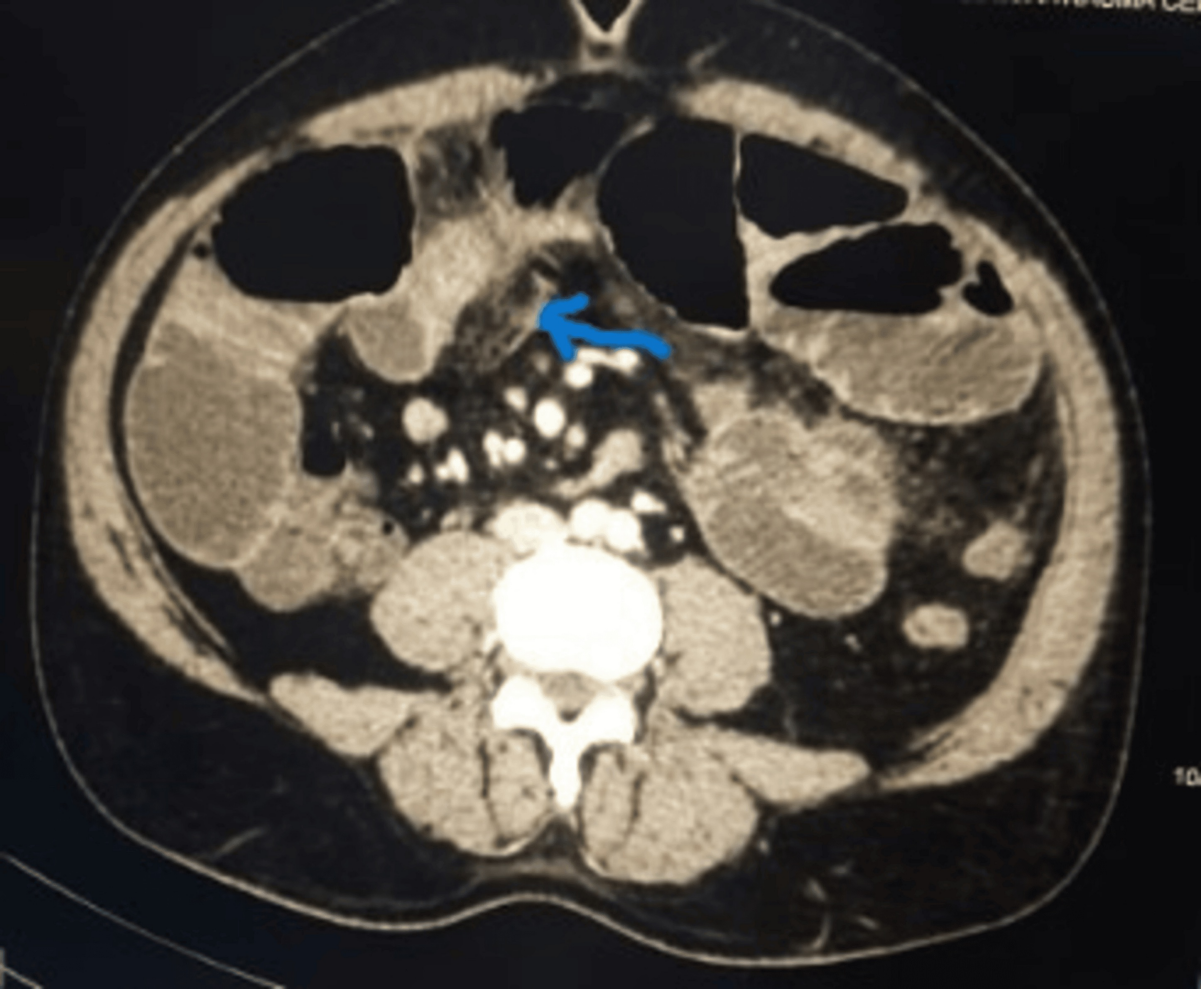
CECT of the abdomen, revealing an inflammatory mass (blue arrow) attached to the anterior abdominal wall, accompanied by adjacent fat stranding and distended small bowel loops that abruptly collapse at the distal jejunum, with minimal peritoneal fluid present CECT: contrast-enhanced computed tomography

Upon arrival, the primary survey of the patient was normal, except for focused Abdominal Sonography in Trauma, which was positive in the left upper quadrant and pelvis. During the secondary survey, the patient exhibited abdominal distention with generalized tenderness, with a nasogastric tube in situ and a daily output of approximately 1,000 mL. The patient underwent a thorough evaluation and a CT scan (Figure 1).

The patient was found to have distended small bowel loops with abrupt collapse at the distal jejunum, accompanied by minimal peritoneal fluid on the CECT of the abdomen. A decision was made to take the patient for exploratory laparotomy and proceed after resuscitation.

**Figure 2:**
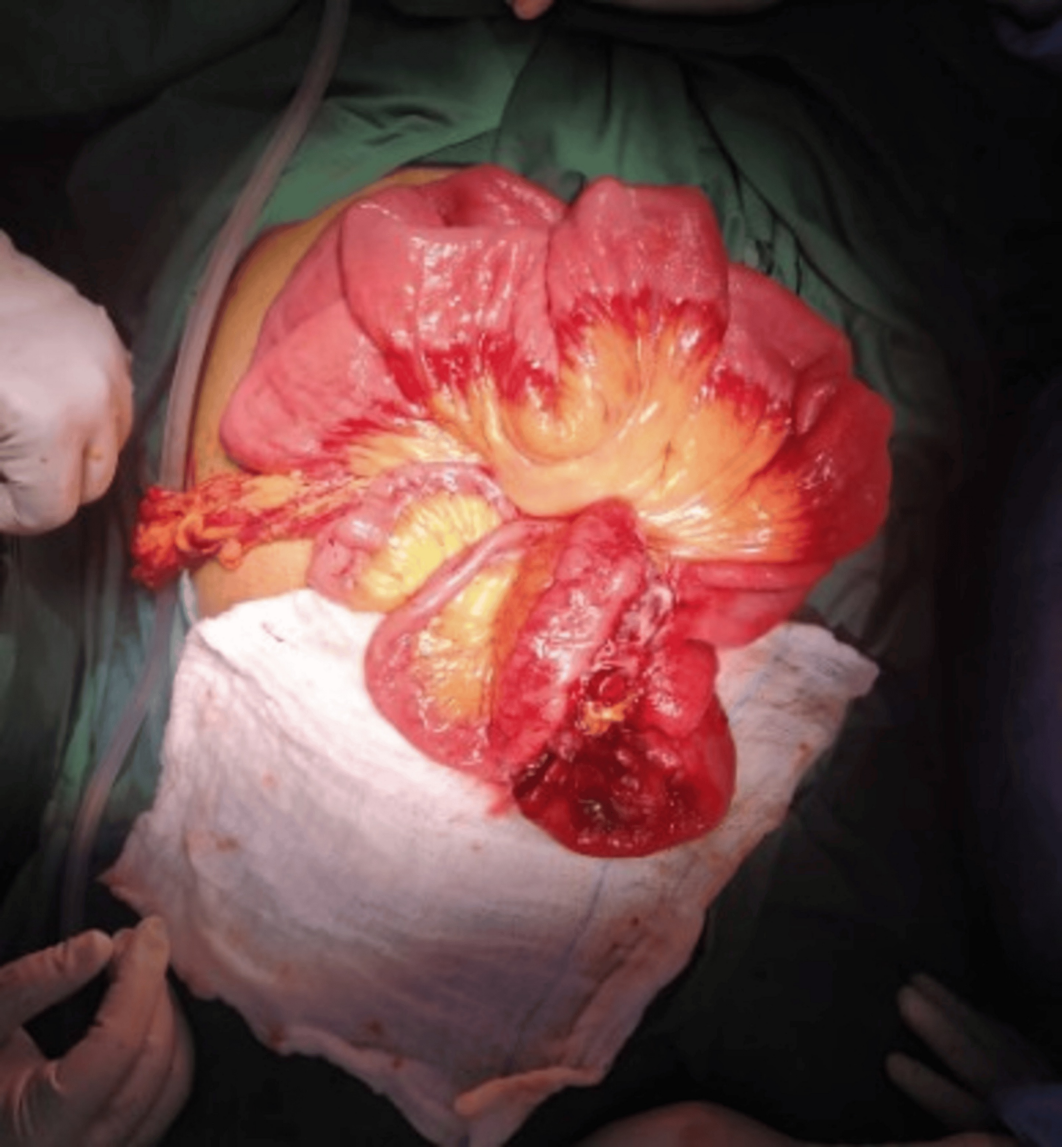
Intraoperative picture, showing an inflammatory bowel mass surrounded by small bowel loops with necrosis of the bowel wall

Intraoperatively, there was a firm mass measuring 10 x 8 cm in the suprapubic region that was densely adherent to the anterior abdominal wall. The mass was surrounded by small bowel loops, making it impossible to separate the small bowel loops from the mass (Figure 2).

**Figure 3:**
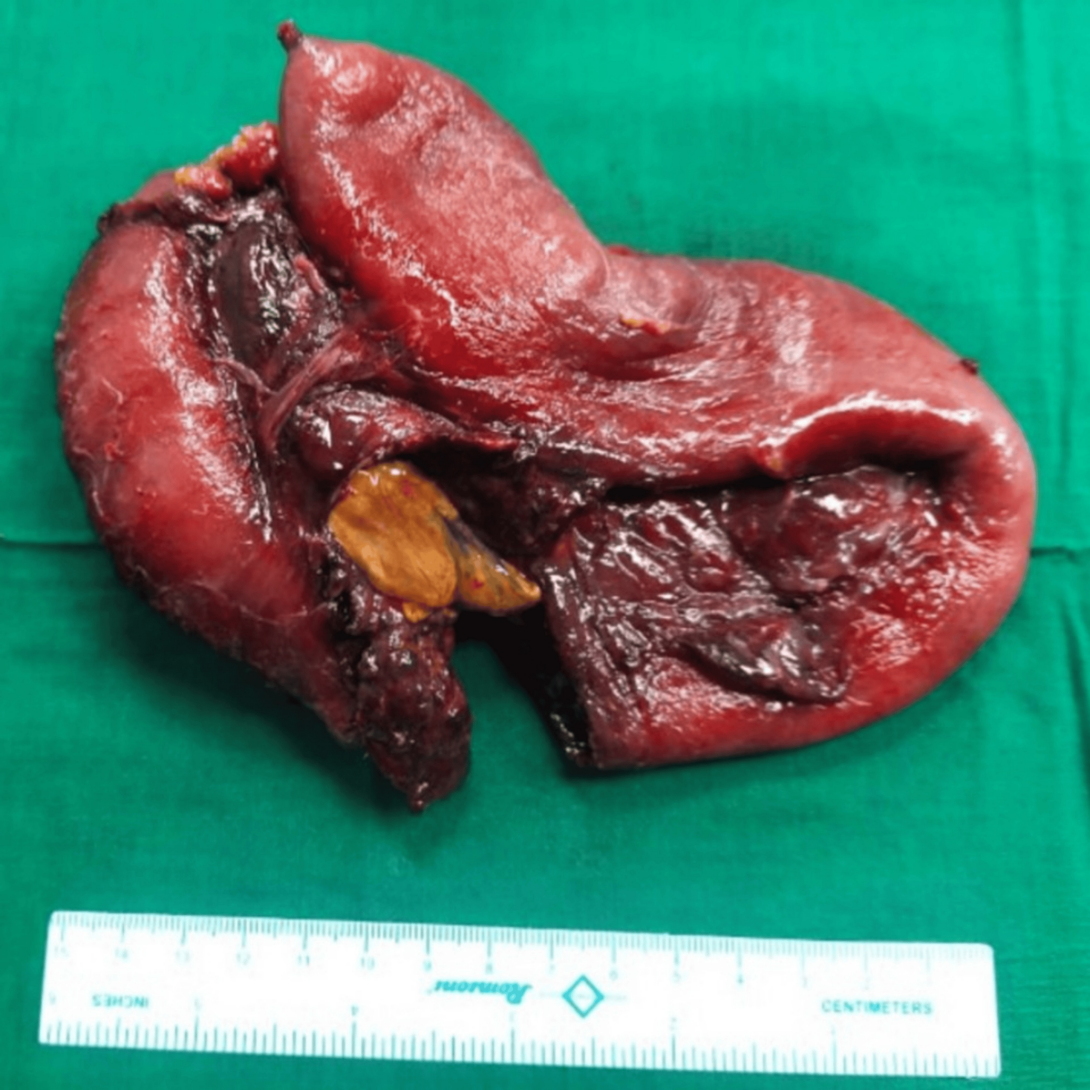
Resected specimen, showing the inflammatory mass with a necrosed bowel wall with adherent surrounding bowel loops

A total mass was resected using a 60 mm Echelon endostapler (Ethicon, Raritan, NJ). The primary side-to-side anastomosis of the cut ends of the distal jejunum was performed using a 60 mm Echelon endostapler (Figure 3).

Peritoneal lavage was performed. An intra-abdominal drain was placed in the pelvis. The rectus sheath was closed using loop nylon no. 1. The skin was left open. The patient was shifted to the ward for postoperative care.

In the postoperative period, the patient experienced abdominal distention with sluggish bowel sounds. Daily nasogastric tube output was 800-900 mL. The patient was managed with IV antibiotics, IV fluids, and analgesics. As we were unable to feed the patient via the enteral route, total parenteral nutrition was started for the patient. On the sixth postoperative day, the patient developed a burst abdomen, for which he was taken to the OR. Intraoperatively, the bowel loops were healthy. A peritoneal lavage was performed, and a mesh laparostomy was created using a Bogota bag (KCI USA, Inc., San Antonio, TX) and a Prolene mesh. The patient developed tachypnea and respiratory distress, necessitating transfer to the ICU. In the ICU, the patient was managed with restricted fluid intake, oxygen supplementation via a facemask, and daily dressing changes for the laparostomy wound. Gradually, the patient improved, passing flatus and stool. The nasogastric tube was removed, and oral feeding was started. The patient underwent split-thickness skin grafting (STSG) over the laparotomy wound eight days after the laparotomy was performed.

STSG uptake was 100%. The patient was discharged from the hospital after the second dressing of STSG, with regular follow-up in the OPD.

## Discussion

BTA is common following road traffic injuries. In addition to road traffic accidents, BTA may also be associated with injuries caused by blunt objects, falls, industrial accidents, blasts, and injuries sustained during sports [6,7]. Small bowel injury resulting from blunt abdominal trauma is an infrequent diagnosis. Early diagnosis and effective management of hollow viscus injury following BTA is very important as it helps in decreasing morbidity and mortality [5,8,9].

In a multicentric study, it was reported that BTA was diagnosed in 1.1% of admitted patients, and small bowel perforation was found in 0.3% of BTA patients. The involvement of multiple organs makes the management of BTA challenging. The most common management protocol is nonoperative management in hemodynamically stable BTA, amounting to 80% of cases, with failure rates of 2%-3%. Features of small bowel injury on CT include free gas or fluid in the peritoneal cavity, fat stranding in the mesentery, or thickened bowel wall enhancement [6,7,10]. Unfortunately, there are cases where no abnormality is found on the initial CT scan. The sensitivity and specificity of CT scans in detecting bowel injury in BTA are 87%-95% and 48%-84%, respectively [11]. Small bowel injuries are missed in 15% of patients during the initial CT scan. Oral contrast during CT scan is not routinely used during the evaluation of BTA [7,12]. Therefore, CT scans are not reliable for detecting blunt small bowel injuries, which leads to difficulties in diagnosis and delayed surgical intervention [5]. Although exploratory laparotomy is a valid option, if expertise and the patient's condition allows, diagnostic laparoscopy is also a valid and less morbid option. In BTA, the diagnosis and management of hollow viscus injury are crucial. Diagnosing hollow viscus injury can be challenging for a surgeon in BTA [8,9].

A male patient in his 40s, a case of BTA following a fall from height, was admitted to the Medical College. Clinically, the patient did not exhibit any features of peritonitis. At the tertiary care center, the CECT abdomen could not identify the small bowel injury. There was a delay in proper diagnosis, and radiological imaging failed to detect the small bowel injury. The patient was managed nonoperatively, and after a significant delay, the patient was referred to a higher center for further management as there was no improvement in the patient's condition. The patient traveled 2,280 km and arrived at a Level I trauma center. Upon admission, the patient was resuscitated. During evaluation, the patient was found to have a small bowel obstruction in the distal jejunum and was taken for exploratory laparotomy.

The highlights of the case were as follows: 1) there was a delay in the diagnosis of small bowel injury in the BTA case, despite the patient being admitted to a tertiary care hospital; 2) there was a delay in referral, even though the patient was not improving with nonoperative management; 3) the delays in diagnosis and management led to increased morbidity, complications, and an extended hospital stay; and 4) the patient has yet to return to a normal routine as they are experiencing a large ventral hernia.

STSG with a planned ventral hernia is an established method for managing a burst abdomen. There are other techniques, such as managing laparostomy with a Bogota bag and Prolene mesh, or using vacuum-assisted closure. In this patient, the bowel was visible in the laparotomy with granulation; hence, the decision to proceed with STSG was taken. In our institute, definitive closure of the ventral hernia is offered when the patient has recovered from the trauma he sustained.

This case report is published to emphasize the importance of clinical examination over radiological imaging in maintaining the differential diagnosis of small bowel injury in BTA patients. The small bowel injury can manifest not only as perforation peritonitis but also as intestinal obstruction. In this case, it is likely a sealed perforation that leads to mass formation and subsequent obstruction. Early diagnosis and treatment are the mainstays of management to avoid further morbidity and complications, as all the complications in this case have occurred due to delays in diagnosis and treatment.

## Conclusions

This case report discusses a male patient in his 40s whose BTA, resulting from a fall from height, unexpectedly presented as an intestinal obstruction. Most probably, an undiagnosed sealed perforation without causing generalized peritonitis converted to inflammatory mass encircling small bowel loops all around and causing intestinal obstruction. This case report teaches us that not all small bowel injuries present as perforation peritonitis. The perforation can go unnoticed, leading to delayed diagnosis, and can present as intestinal obstruction. The delay in diagnosis and treatment leads to further morbidity and complications. The patient should undergo exploratory laparotomy or diagnostic laparoscopy if abdominal signs and symptoms are not settling down with nonoperative management.
